# Ultrathin Platinum Film Hydrogen Sensors with a Twin-T Type Notch Filter Circuit †

**DOI:** 10.3390/s24020548

**Published:** 2024-01-15

**Authors:** Shoki Wakabayashi, Yuki Oh, Haruhito Nakayama, Jin Wang, Toshihiko Kiwa

**Affiliations:** Graduate School of Interdisciplinary Science and Engineering in Health Systems, Okayama University, 3-1-1 Tsushimanaka, Kita-ku, Okayama 700-8530, Japan; pgju8212@s.okayama-u.ac.jp (S.W.); pp737w4n@s.okayama-u.ac.jp (Y.O.); pbu593rx@s.okayama-u.ac.jp (H.N.); wangjin@okayama-u.ac.jp (J.W.)

**Keywords:** hydrogen sensor, ultrathin film, twin-T, notch filter, platinum

## Abstract

In recent years, hydrogen energy has garnered attention as a potential solution for mitigating greenhouse gas emissions. However, concerns regarding the inherent risk of hydrogen gas leakage and potential explosions have necessitated the development of advanced sensors. Within our research group, we have innovated an ultrathin platinum (Pt) film hydrogen sensor that gauges resistance changes in Pt thin films when exposed to hydrogen gas. Notably, the sensitivity of each sensor is contingent upon the thickness of the Pt film. To address the challenge of detecting hydrogen using multiple sensors, we integrated the ultrathin Pt film as a resistance element within a twin-T type notch filter. This filter exhibits a distinctive reduction in output signals at a specific frequency. The frequency properties of the notch filter dynamically alter with changes in the resistance of the Pt film induced by hydrogen exposure. Consequently, the ultrathin Pt film hydrogen sensor monitors output signal variations around the notch frequency, responding to shifts in frequency properties. This innovative approach enables the electrical control of sensor sensitivity by adjusting the operating frequency in proximity to the notch frequency. Additionally, the simultaneous detection of hydrogen by multiple sensors was successfully achieved by interconnecting sensors with distinct notch frequencies in series.

## 1. Introduction

The excessive emission of greenhouse gases from fossil fuel usage is a critical contributor to global warming, causing environmental destruction and abnormal weather patterns. To combat this, renewable energy has emerged as a promising alternative, with hydrogen energy standing out due to its high efficiency and stable supply [1,2,3]. However, hydrogen gas (H_2_) presents safety concerns, given its potential for easy leakage and explosive reactions. Hence, the development of a sensor capable of early H_2_ detection is imperative for ensuring safety.

Various hydrogen sensors [4] are currently available, including metal oxide semiconductors (MOSs) [5,6], catalytic-combustion (CC) types [7,8,9], field-effect transistors (FETs) [10,11,12], and resistance change (RC) types [13,14]. The MOS-type sensor measures resistance changes on MOSs when exposed to H_2_. This sensor has advantages, such as fast response time and high sensitivity at 300 °C. The CC-type sensor measures the temperature change of a catalytic metal film, such as Pt and Pd, when exposed to H_2_. Despite being less influenced by ambient conditions, this sensor typically operates at higher temperatures, resulting in increased energy consumption for heating. In contrast, the FET-type sensor, operating at lower temperatures without a heater, offers lower energy consumption and relatively high sensitivity by measuring the change in the work function of the catalytic metal film on the gate electrode. However, its fabrication process is complex. The RC-type sensor, comprising Pd thin films on a substrate, has a simpler fabrication process than the FET-type sensor. It measures the resistance change of Pd due to H2 absorption, but Pd films may irreversibly degrade due to volume changes during absorption and dissociation of H_2_ [15].

In our research group, we have proposed and developed an ultrathin Pt film hydrogen sensor [16,17,18,19]. Pd films absorb hydrogen molecules and expand, but the state of Pt does not change with H_2_ exposure. Therefore, Pt film sensors are more durable against H_2_ exposure. H_2_ dissociates on the surface of Pt films, injecting electrons into Pt films and causing a decrease in resistance. Several studies have also explored sensors to detect H_2_ using metal-catalyzed gasochromism semiconductors [20]. Similar to those proposed herein, such sensors employ electrons generated by the catalytic reaction. These sensors use semiconductors, which makes it easy to tune their properties. However, those proposed here are based on single films, making their fabrication on polymers easy. Also, two challenges require attention in this sensor. First, variations in sensitivity among sensors arise from slight differences in Pt film thickness, posing a challenge for using multiple sensors in the same environment. Second, employing multiple sensors for hydrogen detection necessitates multiple power supplies and outputs. To overcome these challenges, we have developed a new sensor by incorporating ultrathin Pt films into a twin-T type notch filter circuit [21,22] as resistors. The objective is to create a sensor with adjustable sensitivity and the capability for simultaneous detection by multiple sensors. Additionally, for practical use, we assessed the detection limit of H_2_ and evaluated gas selectivity against H_2_.

## 2. Materials and Methods

**Figure 1:**
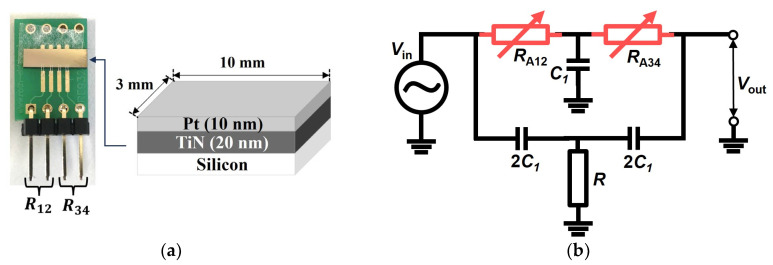
(**a**) The structure of the ultrathin Pt film hydrogen sensor with a length of 3 mm and a width of 10 mm; (**b**) the equivalent circuit of a twin-T; a notch filter circuit combining a low pass filter and a high pass filter in a T-shaped connection.

Figure 1a illustrates the structure of the ultrathin Pt film hydrogen sensor, consisting of three layers: silicon, titanium nitride (TiN), and Pt. Using the sputtering method [23,24] in a vacuum (2.5 × 10^−4^ Pa), TiN and Pt were deposited on a silicon substrate, with thicknesses of 20 nm and 10 nm, respectively. The sensor was bonded to the substrate through a bonding wire. Figure 1b presents the equivalent circuit of the twin-T type notch filter circuit. In this study, the ultrathin Pt film hydrogen sensors were incorporated as resistors (R_12_ and R_34_) into a twin-T type notch filter circuit. This circuit is characterized by a reduction in output signals at a specific frequency, known as the notch frequency ($f_{N}$), which can be determined by

(1) $$f_{N}=\frac{1}{2\pi RC}$$

where *C* is capacitance, and *R* is resistance. 

**Figure 2:**
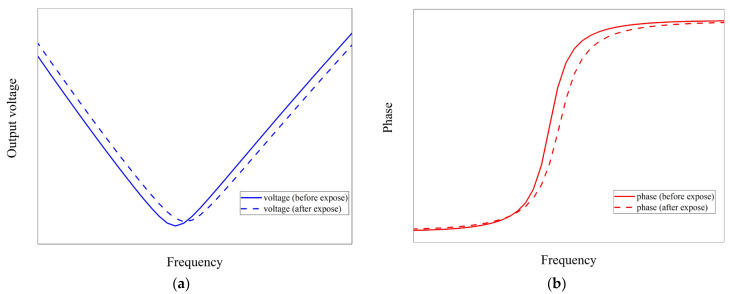
Schematic depicting the frequency dependence of (**a**) amplitude and (**b**) phase before and after exposure to H_2_. The blue graph represents voltage, and the red graph represents phase as a function of frequency. The solid line corresponds to conditions before exposure to H_2_, while the dotted line represents conditions after exposure.

Similar to the earlier-discussed ultrathin Pt film hydrogen sensors, the resistance decreases with increasing H_2_ concentration. Since the Pt film serves as the resistive component of the circuit, the notch frequency shifts upon exposure to H_2_. Figure 2 schematically illustrates the frequency dependence of amplitude and phase before and after H_2_ exposure, showing a slight increase in the notch frequency. Consequently, the concentration of H_2_ can be measured by assessing the amplitude and/or phase of the output signals ($V_{out}$) operating at the frequency ($f_{0}\approx f_{n}$). The sensitivity (*S*) is given by the equation

(2) $$S={\left.\frac{dV_{out}}{df}\right|}_{{f=f}_{n}}$$

Since sensitivity depends on differential properties with frequency, it can be electrically controlled by altering the operating frequency. 

**Figure 3:**
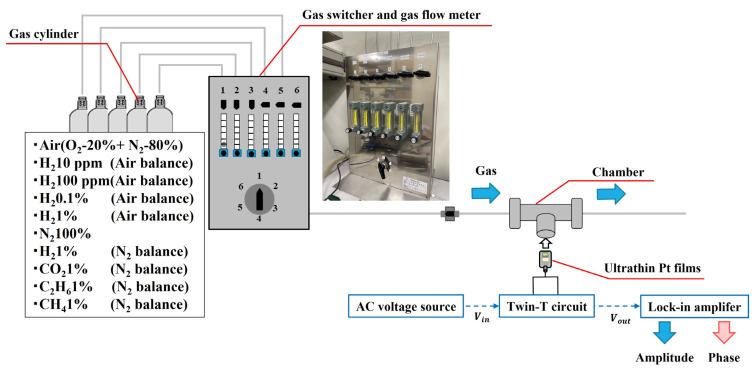
Configuration of the measurement system. Various gases flow from gas cylinders to the chamber through a gas switcher.

The measurement system configuration is shown in Figure 3. Various gases were controlled using a gas switcher, and the flow rate was set to 1 L/min with a gas flow meter. The air gas was balanced by 20% oxygen and 80% nitrogen gas, maintaining a pressure of 1 atm, while H_2_ was balanced by the air gas. Precise H_2_ concentrations were achieved using a gas switcher. The ultrathin Pt film within the chamber was exposed to these gases, and the amplitude and phase of the output signals were measured by applying input from an AC voltage source.

**Figure 4:**
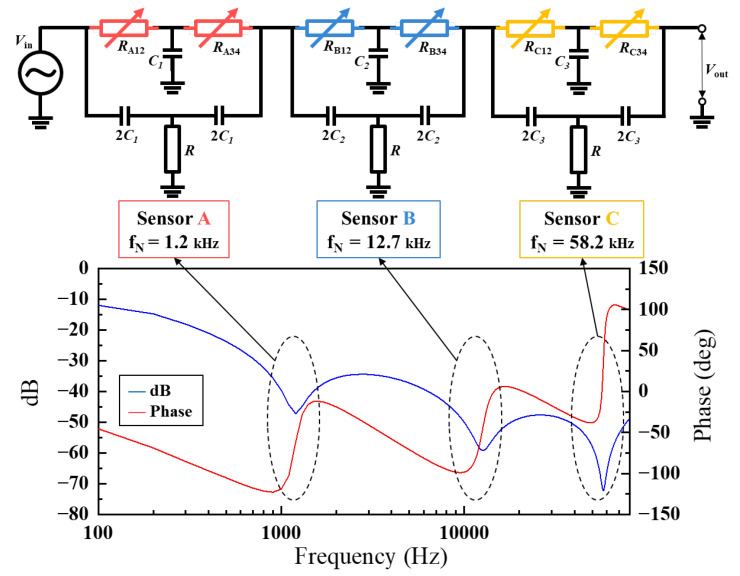
The series circuit of the sensors with different notch frequencies (Sensor A: $f_{N}\;$ = 1.2 kHz, Sensor B: $f_{N}\;$ = 12.7 kHz, Sensor C: $f_{N\;}$ = 58.2 kHz) and the frequency response of the series circuit. The resistance of each ultrathin Pt film (*R*_A_, *R*_B_, and *R*_C_) was nearly the same, allowing each sensor to be designed for different notch frequencies by adjusting the elements in each circuit.

As the sensor operates only at signal inputs near the notch frequency, the operation of multiple sensors is independent of frequency. Thus, simultaneous hydrogen detection by multiple sensors was achieved by connecting three sensors with distinct notch frequencies (Sensor A: $f_{N}$ = 1.2 kHz, Sensor B: $f_{N\;}$ = 12.7 kHz, Sensor C: $f_{N}\;$ = 58.2 kHz) in series for frequency division detection (Figure 4). During 5 min of exposure to air gas and H2, the output voltage was sampled for the composite wave signal of each sensor’s notch frequency. Frequency analyses were conducted using FFT. Additionally, the voltage was repeatedly sampled while the sensor was exposed to air gas and H2, and the power spectrum was measured at each frequency resolution (∆f≅73 [Hz]) in real-time. The number of samples was 4096, and the sampling rate was 30,000 [S/s].

## 3. Results and Discussions

### 3.1. Electrical Control of Sensitivity

**Figure 5:**
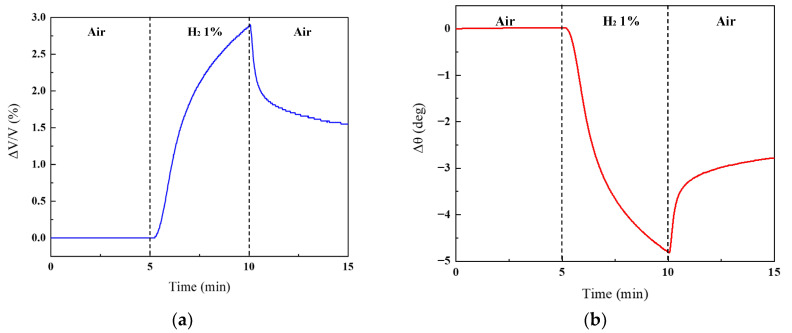
Temporal evolution of (**a**) amplitude and (**b**) phase at the notch frequency of 1.2 kHz. The sensor was exposed to H_2_ for 5 min, with exposure to air gas for an additional 5 min before and after.

In previous investigations, the resistance change of the ultrathin Pt film sensor was quantified, revealing an approximate 1.5% alteration when exposed to H_2_ for 5 min [16]. In this study, the ultrathin Pt film sensor was integrated into a twin-T type notch filter circuit, and the resulting output signals were characterized by measuring both amplitude and phase. The change in amplitude (Δ*V/V*) can be given by

(3) $$∆V/V=\frac{V-V_{0}}{V_{0}}$$

where *V* is the output voltage, and *V_0_* is the voltage at the commencement of the measurement. The phase was initialized at 0 degrees. Figure 5 illustrates the temporal evolution of amplitude and phase (Δ*θ*) at a notch frequency of 1.2 kHz. The changes in amplitude and phase were 2.8% and 4.8 degrees, respectively. Furthermore, the change in output signals from the sensor with the twin-T circuit was found to be 1.3% to 2.3% higher compared to the previous sensor. After H_2_ exposure, the sensor exhibited a long recovery time: it took more than 12 h for the output signals to recover and return to their original value after the gas supply switched from H_2_ to air. To overcome this problem, the sensor was subjected to thermal treatment by pulsed current injection [18].

**Figure 6:**
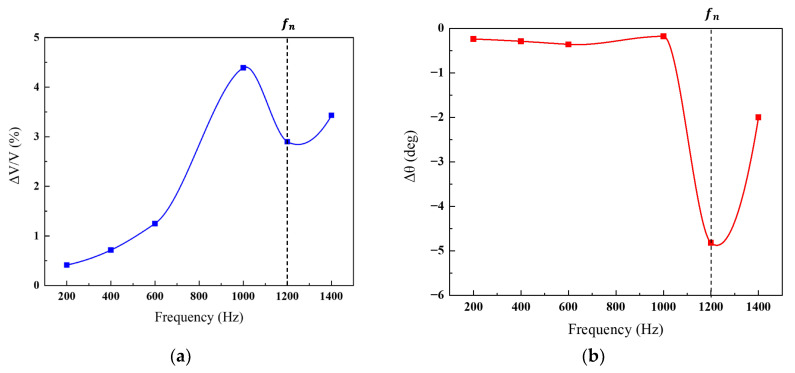
Variation in (**a**) amplitude and (**b**) phase concerning the operating frequency of the circuit when the sensor was exposed to H_2_.

Figure 6 illustrates the amplitude and phase variation as a function of the circuit’s operating frequency when exposed to H_2_. The measurements involved adjusting the frequency in increments of 200 Hz, ranging from 200 Hz to 1400 Hz. The most significant change in amplitude occurred at 1.0 kHz, reaching 4.3%, while at the notch frequency of 1.2 kHz, it measured 2.9%. The amplitude increase at frequencies deviating from 1.2 kHz is attributed to the sensitivity (*S*) rise. However, excessive deviation from the notch frequency may prevent adequate signal attenuation by the twin-T circuit, resulting in reduced amplitude change. The maximum phase change was observed at 1.2 kHz, registering at 4.8 degrees, diminishing as the operating frequency moved away from the notch frequency. By finely tuning the operating frequency near the notch frequency, the changes of both the amplitude (2.9–4.3%) and phase (2 degrees to 4.8 degrees) could be adjusted. This outcome distinctly highlights the electrical regulation of sensor sensitivity through operating frequency manipulation.

**Figure 7:**
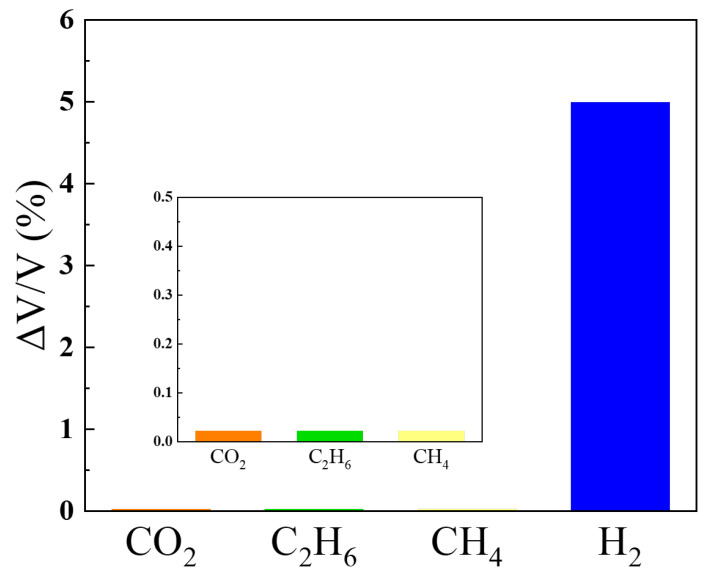
Gas selectivity when exposed to 1% nitrogen-based H_2_ and interfering gases for 5 min.

Figure 7 presents the gas selectivity under exposure to 1% nitrogen-based H_2_ and interfering gases for 5 min. Carbon dioxide, methane, and ethane served as interfering gases. Carbon monoxide and nitrogen oxides will also be explored in future studies. The change in H_2_ exposure was approximately 5%, whereas the exposure to interfering gases changed by only 0.02%, constituting less than one-hundredth of the change in H_2_ exposure. The sensor developed in this study operates at room temperature and remains unaffected by interfering gases, exhibiting selective reactivity exclusively with H_2_. 

**Figure 8:**
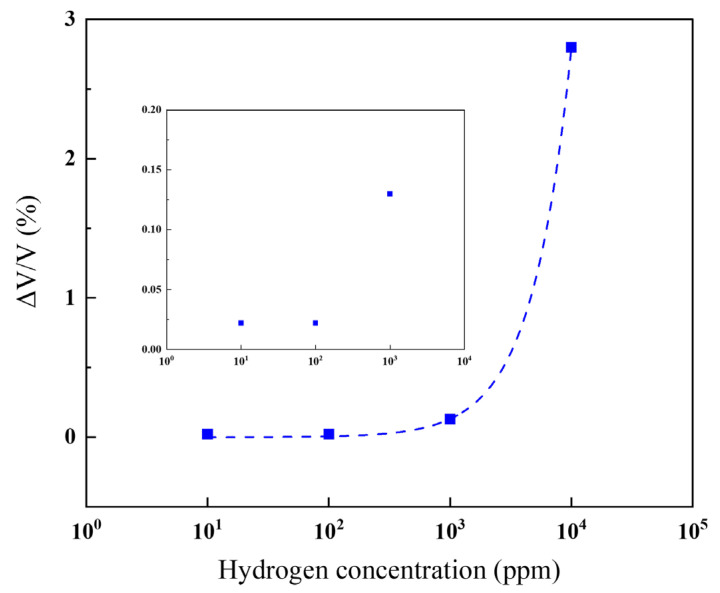
Change in amplitude when the hydrogen concentration was varied by one digit.

Figure 8 depicts the detection limit for hydrogen concentration by varying the concentration by one digit. The results demonstrate a response to hydrogen concentrations as low as about 10 ppm. The change in amplitude increased with rising hydrogen concentration, measuring 0.02% at 10 ppm and 0.14% at 1000 ppm. Considering the lower explosive limit concentration of H_2_ in the air is 4%, the sensor developed in this study proves capable of detecting leaks before reaching hazardous levels.

### 3.2. Simultaneous Hydrogen Detection

**Figure 9:**
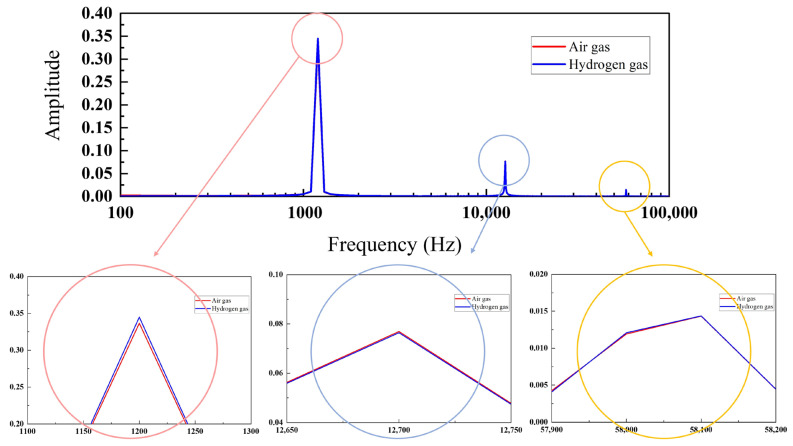
Results of FFT analysis on the output signal of the composite wave input to the series circuit when air gas and H_2_ flowed into Sensor A.

Figure 9 presents the results of FFT analysis on the output signal of the composite wave input to the series circuit when air gas and H2 flowed into Sensor A. The change in amplitude at 1.2 kHz was 2.45%, while changes at 12.7 kHz and 58.1 kHz were 0.68% and 0.21%, respectively. Thus, the amplitude increased only at the operating frequency of Sensor A with hydrogen exposure. Table 1 details the change in amplitude at each frequency when H_2_ flowed into each sensor. The amplitude at the operating frequency of the sensor changed the most with the inflow of H_2_, suggesting that the ultrathin Pt film hydrogen sensor with a twin-T type notch filter could be employed in a series circuit for simultaneous hydrogen detection by multiple sensors.

**Figure 10:**
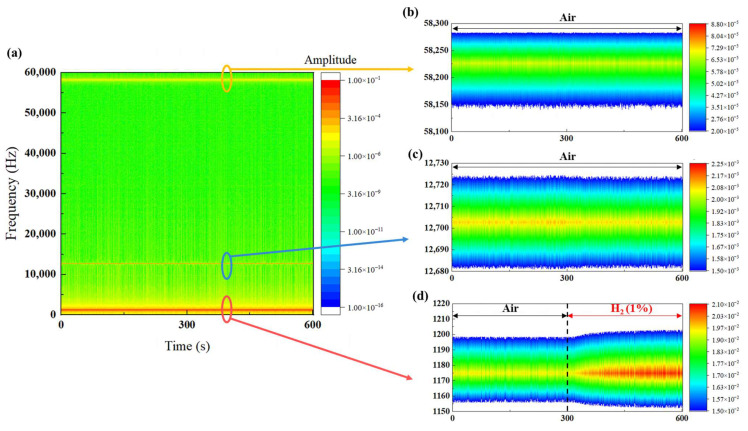
(**a**) Spectrograms when Sensor A was exposed to H_2_; (**b**) enlarged view around 1.2 kHz; (**c**) enlarged view around 12.7 kHz; and (**d**) enlarged view around 58.2 kHz.

Figure 10a displays the spectrograms when Sensor A was exposed to H_2_. The horizontal axis represents time, the vertical axis indicates frequency, and the color code represents amplitude. Amplitudes at the frequency bands around 1.2, 12.7, and 58.2 kHz were larger than those at other frequencies. Figure 10b provides an enlarged view around 1.2 kHz. The amplitude remained constant from 0 to 300 s during exposure to the air gas. In contrast, the amplitude increased from 1.9 5 × 10^−2^ to 2.10 × 10^−2^ at 1175 Hz during 300–600 s when exposed to H_2_. This result indicates that Sensor A detected H_2_, and the hydrogen detection using this sensor could be visualized. On the other hand, the amplitude at the frequency bands around 12.7 kHz (Figure 10c) and around 58.2 kHz (Figure 10d) did not change as they were not exposed to H_2_. Only the amplitude of Sensor A exposed to H_2_ changed. Simultaneous hydrogen detection by multiple sensors in real-time is possible, making it easier to locate leaks.

## 4. Conclusions

We developed a resistance change-type sensor utilizing an ultrathin Pt film. In this paper, a twin-T type notch filter sensor incorporating the ultrathin Pt film as a resistive element was fabricated. The objective was to adjust the sensitivity of the sensor and enable the simultaneous detection of H_2_ by multiple sensors. Designing multiple sensors with consistent sensitivity allows their use in the same environment. Moreover, by connecting twin-T-type notch filter sensors in series, the operation of each sensor is synchronized, leading to the creation of a system that functions only when the sensor is exposed to H_2_. This system would facilitate the safe handling of hydrogen in large-scale situations.

In future work, we aim to expand the number of series circuits to achieve multipoint simultaneous measurements through frequency multiplexing.

## Figures and Tables

**Table 1:** Change in amplitude at each frequency when H_2_ flew into each sensor.

Sensors Exposed to H_2_	1.2 kHz	12.7 kHz	58.2 kHz
Sensor A	2.45%	0.68%	0.21%
Sensor B	0.17%	2.61%	0.81%
Sensor C	0.19%	0.10%	17.2%

## Data Availability

The data that support the findings of this study are available from the corresponding author, [T.K.], upon reasonable request.
